# How do inner screens enable imaginative experience? Applying the free-energy principle directly to the study of conscious experience

**DOI:** 10.1093/nc/niaf009

**Published:** 2025-04-22

**Authors:** Chris Fields, Mahault Albarracin, Karl Friston, Alex Kiefer, Maxwell J.D Ramstead, Adam Safron

**Affiliations:** Allen Discovery Center, Tufts University, Medford, MA, United States; VERSES, Los Angeles, CA, United States; Département d’informatique, Université du Québec à Montréal, 201, Avenue du Président-Kennedy, Montréal, Canada; VERSES, Los Angeles, CA, United States; Queen Square Institute of Neurology, University College London, Queen Square, London, United Kingdom; VERSES, Los Angeles, CA, United States; Monash University, Wellington, Clayton, Australia; Queen Square Institute of Neurology, University College London, Queen Square, London, United Kingdom; Allen Discovery Center, Tufts University, Medford, MA, United States; Department of Psychiatry & Behavioral Sciences, Johns Hopkins University, School of Medicine, Baltimore, MD, United States; Institute for Advanced Consciousness Studies, Santa Monica, CA, United States

**Keywords:** Aphantasia, Cognitive architecture, Depression, Inner speech, Introspection, Metacognition, Planning, Visual imagery

## Abstract

This paper examines the constraints that the free-energy principle (FEP) places on possible model of consciousness, particularly models of attentional control and imaginative experiences, including episodic memory and planning. We first rehearse the classical and quantum formulations of the FEP, focusing on their application to multi-component systems, in which only some components interact directly with the external environment. In particular, we discuss the role of internal boundaries that have the structure of Markov blankets, and hence function as classical information channels between components. We then show how this formal structure supports models of attentional control and imaginative experience, with a focus on (i) how imaginative experience can employ the spatio-temporal and object-recognition reference frames employed in ordinary, non-imaginative experience and (ii) how imaginative experience can be internally generated but still surprising. We conclude by discussing the implementation, phenomenology, and phylogeny of imaginative experience, and the implications of the large state and trait variability of imaginative experience in humans.

## Highlights

- We present a model of imaginative experience that is compliant with the free-energy principle (FEP).
- We particularly address the questions of how imaginative experience is controlled and how it can be surprising.
- We emphasize the roles of thermodynamic energy flows and metacognitive control in regulating both imaginative and non-imaginative experience.
- We discuss the implementation, phenomenology, and phylogenetic distribution of imaginative experience, as well as state and trait variability in imaginative experience in humans.

## Introduction

This paper investigates the constraints placed on theories of consciousness by the variational free-energy principle (FEP). The FEP is concerned with dynamical systems having state spaces that can be partitioned, over some time period of interest, into the states of some “thing,” “particle,” or subsystem of interest, and the states of the “environment” of that subsystem, which compose (by definition) the rest of total state space (Friston, 2019). Such partitioning is possible if, but only if, both the “particle” or “thing” and its “environment” have statistical boundaries or Markov blankets, and behave in ways that preserve their distinguishability or mutual conditional independence, and hence their boundaries, over the entire time period of interest. Under these conditions, which correspond to conditions of weak interaction or sparse coupling, both the “thing” and its “environment” have “internal” states not directly perturbed by their interaction. The FEP provides, effectively, a dictionary for translating descriptions of the behavior of dynamical systems equipped with such partitions stated in the languages of either classical (Friston, 2019; Ramstead *et al.*, 2022) or quantum (Fields *et al.*, 2022) physics into the language of approximate Bayesian inference, i.e. Bayesian satisficing. In particular, whenever a “thing” and its “environment” behave in a way that preserves their mutual boundary and hence their distinguishability, the dynamics of their respective internal states can be described as implementing models (Safron, 2020) of each others’ behavior that generate, and then test, predictions of each others’ actions, where the “action” of—informational output from—each system on the boundary of the other constitutes the “sensation” of—informational input to—the other system.^1^

The FEP does not stipulate any particular definition of the term “consciousness” and is not, in and of itself, a theory of consciousness (Hohwy and Seth, 2020). However, since the FEP provides a principled inferential description of all systems that can be distinguished from their environments in terms of both internal information processing and input/output behavior, it constrains theories of consciousness indirectly, by attributing the minimal structures (e.g. perceptual inferences) that are necessary for consciousness in paradigmatically conscious systems—including all organisms—and more broadly to all physical systems sparsely coupled to their environments. It characterizes systems that both modify their behavior in response to environmental inputs and modify their environments by acting on them as *agents* (Safron, 2020; Safron, 2021a; Friston *et al.*, 2022), indeed as Bayesian agents, with the degree of agency correlated with both the representational power and the temporal depth of the deployed model (Friston *et al.*, 2022). This agentic perspective on physical systems may appear counter-intuitive, but as shown by the Conway-Kochen “free will” theorems (Conway and Kochen, 2006; Conway and Kochen, 2009), viewing all physically implemented systems as “free” in the specific sense of exhibiting behaviors not causally determined by their environments is required for consistency with the combination of non-superdeterminist quantum theory and special relativity; see Fields and Levin (2025) for further discussion.^2^

By treating dynamical systems as agents and interpreting physical interactions between them as sensation-action loops mediated by internal, model-based Bayesian inference, the FEP suggests a simple constraint on theories of sensation, and hence on theories of sensory consciousness: it suggests that systems “experience” whatever is “written” on their boundaries (Fields *et al.*, 2021). It is important to emphasize that “boundaries” here are *informational* boundaries—boundaries in state spaces—that may not, and in general do not, coincide with intuitively-obvious spatial boundaries such as cell membranes or the skin of an organism. “Sensation” includes whatever is written by the action of the environment upon the informational boundary, but also, potentially, includes whatever may be written by the action of internal processes on the informational boundary as discussed in detail below. While the classical formulation of the FEP is silent on what else a system may be capable of experiencing, the quantum formulation imposes a further constraint on any experiences with specific content. In the quantum formulation, classical information—information with specific content—is encoded *only* on informational boundaries, as shown explicitly in Fields *et al.* (2022). The quantum formulation makes up for this by inducing such boundaries between any systems that are not entangled (Fields, 2024); hence the quantum FEP requires internal boundaries in any systems that are not pure quantum computers, i.e. any systems with distinct, mutually conditionally-independent components. Hence the quantum formulation can be read as rendering all experience with specific content “sensory” as this term is used in the FEP (Friston *et al.*, 2020), and as associating such sensory experience with all boundaries between mutually conditionally-independent systems.^3^ The quantum formulation of the FEP is fully scale-free and applies in the same form to all physical systems; indeed it follows directly from the quantization of information into discrete units (Fields *et al.*, 2022). When information flows between systems—i.e. causal influences—are large enough to be treated as continuous, one obtains the classical formulation, which is effectively a low-resolution or “macroscopic” approximation. It bears emphasis that neither formulation of the FEP says anything directly about the semantics of these experiences or “what they are like” for the system that has them. From a biological perspective it is natural, but by no means logically necessary, to associate prediction errors with negative valence and hence stress (Peters *et al.*, 2017; Linson *et al.*, 2020; Fields *et al.*, 2021; Hesp *et al.*, 2021).

Identifying “experience” with input to a Markov blanket raises, however, an obvious problem when applied to complex organisms, particularly humans: it appears to restrict awareness to awareness of the external environment, so while it is consistent with humans or other systems engaging in complex cognitive tasks such as planning, it appears to rule out any *experiences* of the performance of such tasks. Indeed, it appears to rule out *all* imaginative (i.e. counterfactual) experiences, including mnemonic experiences or planning as well as dreams, introspective thought, etc. Such experiences involve sensations, such as the aural sensation of inner speech, the visual sensation of self-generated imagery, or the kinesthetic sensation of imagined movement (Kosslyn *et al.*, 2001), but the causal source of these sensations is not the external environment or even, as in somatosensory or kinesthetic sensations, the body below the brainstem. As pointed out in Fields *et al.* (2021), preserving the equation of “experience” with input crossing, and hence being encoded on, a boundary requires postulating the existence, in any systems that have imaginative experiences, of internal informational boundaries, or “inner screens” on which such “inner” experiences can be encoded. This raises an obvious question: given some system *S* that is capable of imagination, are *S*’s experiences—as distinct from the potential experiences of any proper components of *S*—written on one informational boundary or on two or more? If the answer is two or more, how can this be consistent with the experiences written on these multiple boundaries all being the experiences of *S*, not the experiences of multiple distinct systems smaller than *S*? These are questions that any inner-screen theory must address.

The idea of experiences as encoded on “inner screens” is, of course, far from novel; an internal “theater of consciousness” is postulated explicitly in Baars’ (2005) global workspace theory (GWT) and is implicit in traditional thinking about consciousness at least back to Descartes. It is not implausible to regard the “mind” or “inner world” of folk psychology as some kind of inner screen. From an informal, intuitive perspective, imagination plays a large role in the notion of “mental life” and would seem to be a hallmark of consciousness; the phrase “unexperienced inner speech,” for example, sounds like an oxymoron. Our goal in the present paper is to determine whether, and if so how, these informal ideas can be made more rigorous via the FEP, and in particular, whether they can be made strictly compliant with a requirement that all the experiences of a system *S* are encoded on *S*’s informational boundary. As noted earlier, the FEP is not itself a theory of consciousness, and says nothing about whether any of the internal information processing that it characterizes agents as doing—for example, the computation of expected free energies for the outcomes of alternative policies during planning—is done consciously. Hence our goal here is to specify necessary conditions for *imaginative* experiences in systems that meet previously-proposed necessary conditions for *any* experiences, e.g. the condition of temporal coarse-graining proposed by Whyte *et al.* (2024). We will focus, in particular, on three questions relevant to any “inner screen” construction. The first is whether, and if so how, an FEP-compliant architecture can support experienced metacognitive control, including experienced attentional control via the top-down modulation of Bayesian precision. While it is clear that generic hierarchical predictive-coding models can support attentional control, it is not clear what constraints, if any, such models generically place on the *experience* of exercising such control, either at the whole-agent level or at the level(s) of the components. Recent models proposed by Bennett *et al.* (2024) and Laukkonen and Chandaria (2024), for example, incorporate hierarchical predictive coding with attentional control without, apparently, localizing the experience of attentional control to any boundary, whether external or internal. The second question is how imaginative experience can employ the same spatio-temporal and object-recognition reference frames—concepts, categories, properties, coordinate systems and so forth—employed in ordinary, non-imaginative experience (Kosslyn *et al.*, 2001). When faced with the “experienced by whom?” question, traditional inner-screen models postulate an “inner observer” possessing whatever conceptual structure is required to interpret what is “displayed” on the inner screen. As pointed out by Maturana and Varela (1980) and many others, any model in which the inner observer must duplicate the conceptual structure attributed to the whole observer is circular and therefore non-explanatory. An acceptable, FEP-compliant model of imaginative experience must show how the agent of interest can be the “experiencer” of both externally- and internally-generated sensations and the “actor” for both externally- and internally-directed actions while employing the same physically-implemented conceptual structure, acting on the same informational boundary, for both. It must also explain how some agents, including most humans, can switch between deliberate actions accompanied by imaginative experiences—e.g. “thinking” via inner speech or visualization—and similar actions performed “automatically” in the absence of such imaginative experiences, e.g. well-practiced actions performed in “flow” states. The third question is how, given that they are internally generated, imaginative experiences can be surprising, as they so often are, and as they must be in order to be cognitively useful.

Ours is, of course, not the first attempt to develop an integrative treatment of consciousness that is compliant with the FEP; for previous general discussions, see e.g. Whyte *et al.* (2022) or Wiese and Metzinger (2017). One notable attempt to integrate a broad spectrum of theories of consciousness is integrated world modeling theory (IWMT) (Safron, 2020; Safron, 2022). IWMT represents an attempt to integrate FEP accounts with two other leading theories of consciousness, namely, GWT and integrated information theory (IIT 3.0) (Tononi, 2015; Tononi *et al.*, 2016). A second body of work that has focused on the role of active inference in the selection of “winning hypotheses” corresponding to conscious perceptual contents, as in binocular rivalry paradigms (Hohwy, 2013; Hohwy, 2022; Hohwy *et al.*, 2008; Parr *et al.*, 2019), has also presented an FEP-theoretic perspective on lines of evidence motivating many distinct theories of consciousness. More recently, Whyte *et al.* (2024) have argued that the active inference framework can be interpreted as setting constraints on any theory of consciousness. One of these is that what is “experienced” by any system is (a subset of) its posterior probability distributions, a requirement that is consistent with what we outline below provided encodings on MBs are taken to be coarse-grained. The present proposal differs from this previous work in taking an explicitly architectural approach that considers each component of a complex cognitive agent to be an agent that must cope with its own immediate environment, which in most cases consists primarily or even exclusively of other components of the complex agent. It aims to show how active inference at the scale of the complex agent as a whole can be re-described as the collective behavior of its component agents, and hence to show how the experience of the complex agent as a whole relates to the experiences of its component agents. This work can, therefore, be considered to represent a step toward identifying necessary, though not demonstrably sufficient, conditions for the realization of imaginative consciousness in any entity to which such experience might be attributed.

The paper is structured as follows. We first briefly review the FEP in both its classical and quantum formulations, and develop an explicit formal framework, applicable to arbitrary systems, for specifying perception and action capabilities and the representation of space and time. We also provide an explicit representation of the flow of thermodynamic free energy (in living systems, metabolism), and of how systems employ environmentally-sourced thermodynamic free energy to power cognitive processing. We then provide a minimal model of an active inference agent with multimodal perception and action that employs a metacognitive controller to differentially allocate thermodynamic resources and attention to different ways of interacting with its environment. This system is capable of planning—i.e. of computing expected free energies and algorithmically choosing a policy with minimal expected free energy (EFE)—and hence meets the criteria for consciousness suggested by Whyte *et al.* (2024). However, it would have no imaginative experiences associated with this cognitive activity; it would experience its behavior in the way that a person with complete, multi-modal aphantasia (Zeman, 2024) might experience it. We then develop a minimal extension of this model that is capable of imaginative experiences. We discuss how the differences between these two models allow us to capture the range of variation of both state and trait human imaginative experiences. We conclude with a discussion of core concepts and predictions that emerge from this treatment.

## Preliminaries: a short introduction to the free-energy principle

### The classical formulation of the free-energy principle

The FEP is a mathematical principle that, much like the principle of least action, can be used to derive the mechanics of dynamical systems, i.e. equations of motion that describe their observable dynamics (Friston, 2019; Ramstead *et al.*, 2023; Ramstead *et al.*, 2024). Just as one can think of the principle of least action as the variational principle that underwrites classical mechanics, one can think of the FEP as the variational principle that describes the way that probabilistic beliefs evolve over time; which is known as Bayesian mechanics (Ramstead *et al.*, 2023; Sakthivadivel, 2022). The FEP provides an explanation—from first principles—of why any thing that physically exists looks as if it infers the properties of the environment to which it is coupled, but from which it can be separated (Ramstead *et al.*, 2023). In other words, the FEP provides us with a way to model the dynamics of any physical “thing” that exists, as itself modeling the statistical structure of the other “things” that constitute its embedding environment (Ramstead *et al.*, 2024).

**Figure 1.**
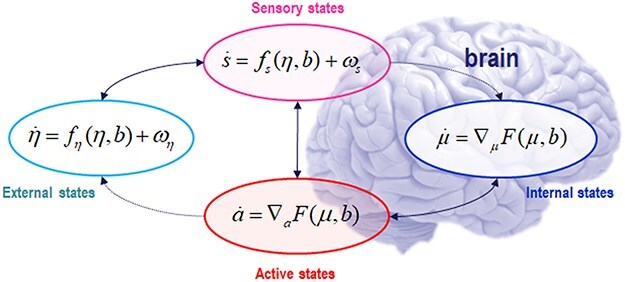
Illustration of a Markov blanket (MB). *Internal* and *external* (i.e. environmental) states (denoted *η* and *µ*, respectively) interact via *sensory* and *active* blanket states (denoted **s** and **a**, respectively, with the total blanket state denoted **b** = (**s**,**a**)); the latter induce a conditional independence between internal and external states. In virtue of this conditional independence, one can associate internal states with the parameters or sufficient statistics of a conditional (a.k.a., variational) density over external states, given blanket states. In turn, one can then interpret internal (and active) dynamics as a gradient flow on a variational free energy that plays the role of a marginal likelihood or model evidence in statistical inference. Equations represent time derivatives (the notation $\dot{\mathbf{a}} = \partial \mathbf{a}/\partial t$; $\nabla$ is the gradient operator) of the state variables as functions of each other. The vertical solid arrow emphasizes that the time evolution of both sensory and active states depends on the total blanket state **b**.

The core construct of the FEP formulation is the *Markov blanket* (MB), which separates a “thing” or particle from its environment—but also couples the one to the other (Ramstead *et al.*, 2023; Ramstead *et al.*, 2024; Sakthivadivel, 2022). An MB renders the internal states of some thing conditionally independent from the external states of that which it is not. Analyses of systems in terms of active inference (Parr *et al.*, 2022), which applies the FEP to the study of action-perception loops, assume a further decomposition of the MB into sensory and active states (Palacios *et al.*, 2020). By construction, sensory states are those that influence but are not influenced by internal states; whereas, symmetrically and reciprocally, active states influence but are not influenced by external states. Figure 1 illustrates this.

Heuristically, the FEP says that, if a system can be partitioned into “things” that have MBs, then those “things” will look as if they encode and update probabilistic beliefs about the other “things” to which they are coupled. More precisely, the FEP says that, when they exist, the Markov blanketed subsets of a random dynamical system will look as if they infer or estimate or “track” the parameters of probability distributions over other subsets of the system. This is because the dynamics or observable behavior of any Markov blanketed “thing” can be read as a gradient flow over surprise and because anything that flows over surprise gradients is, mathematically, a form of inference (Ramstead *et al.*, 2024). In this setting, we can express the dynamics of active and internal states of a “thing” in terms of a gradient flow over surprise or (equivalently) on an upper bound of surprise, known as variational free energy. This is a tractable quantity that depends upon the data generated by sensory states, and on a generative model of how external states cause sensory states (Friston, 2010); where the word “cause” is used in the straightforward (dynamical systems) sense that one state causes changes in another state because the first state enters the equations of motion of the second (Ramstead *et al.*, 2023). Effectively, this allows one to interpret the autonomous (i.e. internal and active) dynamics as a process of inference; namely, changing in a way that minimizes variational free energy or, equivalently, maximizing the evidence for the generative model. This is sometimes called self-evidencing (Hohwy, 2016).

In a system that conforms to the FEP, the internal states of a particle will look as if they are updating a probabilistic representation of the causes of its sensory states. Note that this description can never be directly verified, because the internal states are inaccessible, provided the MB remains intact: just as the system observes its environment only via its blanket, the environment observes the system only via its blanket.^4^ This probabilistic representation, parameterized by internal states, is a variational density over external states. Particles model their environments via the evolution of this variational density. It is worth noting that this sense of “representation” differs substantially from other usages of this term in the literature, particularly the idea of “representation” as causal-informational dependency that features in traditional philosophical psycho-semantics (Dretske, 1981; Fodor, 1990; Usher, 2001) and has been roundly criticized by Maturana and Varela (1980) among others. Broadly speaking, Dretske (1981), Fodor (1990) and others posit atomic relations of lawful covariation between the internal states of agents, on the one hand, and entities or events in the represented world (what a FEP theorist would call external states), on the other. On this account, an internal state represents an external state if, and only if, there exist lawful relations of covariation between both states, such that changes in internal states track changes in external states. In the FEP formulation, the internal states of a system do not *directly* track or covary with the external states of a system: rather, they encode the parameters of a conditional *probability density* over external states—the variational density—and it is this density which represents external states (Sprevak, 2020). This is a core but crucial difference. That said, this form of mapping relates to “tracking” in the conventional sense, since internal paths of least action encode expectations about external paths (where action is the path integral of variational free energy). Crucially, the factors of the conditional density (e.g. types of objects, outcomes, and events) that “carve up” the world may vary arbitrarily, subject only to the constraint that model evidence is conserved on average (Ramstead *et al.*, 2023; Sakthivadivel, 2022). This account of representation may thus be regarded as justifying (or dovetailing with) Quine’s view that categories—in terms of which we experience the world—are akin to theoretical posits, which earn their keep via their predictive and explanatory power with respect to sensation (Quine, 1960). Despite this, structural correspondences can be expected to obtain at the level of internal and external states as a whole (Conant and Ashby, 1970; Gładziejewski, 2016; Kiefer and Hohwy, 2018; Ramstead *et al.*, 2020b), where the KL-divergence between the variational and generative densities has been argued to provide an account of misrepresentation, and thus a form of semantic normativity (Kiefer and Hohwy, 2018; Ramstead *et al.*, 2020a).

### The quantum theoretic formulation of the free-energy principle

As mentioned in the Introduction, the continuous representation of causal processes, and hence of information flows between systems, employed in the classical formulation of the FEP can be viewed as a macroscopic limit of a discrete representation. If information is quantized into discrete units—e.g. bits—a quantum formulation of information flow is required. Standard quantum theory provides such a representation (Nielsen and Chuang, 2000). Formulating the FEP in the language of quantum theory extends its range of application to systems not ordinarily thought of as “things,” e.g. to quantum fields. For the present purposes, the quantum formulation provides both the strong constraint that classical information must be localized to boundaries discussed above, and a convenient language—the language of quantum reference frames (Bartlett *et al.*, 2007) described in § 2.3 below—with which to characterize sensation-action loops with their associated predictive-model components as computational modules.

Similar to the classical formulation of the FEP that starts with a random dynamical system, the quantum formulation starts with a quantum process $\mathcal{P}_U(t)$ defined on a finite, isolated system *U* that is taken to include “everything of interest” in some situation. The process $\mathcal{P}_U(t)$ for an isolated system can be written as $\mathcal{P}_U(t) = exp((-\imath/\hbar)H_U(t))$, where $\hbar$ is the reduced Planck’s constant and *H_U_* is the Hamiltonian or total energy operator defined on the state (Hilbert) space $\mathcal{H}_U$ of *U*. Any such system *U* can be decomposed into arbitrarily-chosen components *S* and *E*, a “thing” and its “environment” respectively, by writing $\mathcal{H}_U = \mathcal{H}_S \otimes \mathcal{H}_E$, where ⊗ is the Hilbert-space tensor product, and $H_U = H_S + H_E + H_{SE}$, where *H_U_*, *H_S_*, and *H_E_* are the internal Hamiltonians of *U*, *S*, and *E* respectively, and *H*_*SE*_ specifies the interaction between *S* and *E*. Provided that the coupling *H*_*SE*_ between the thing and environment is sufficiently weak, the joint state $|U\rangle$ (employing Dirac’s notation) may be (approximately) separable, i.e. not (significantly) entangled, and hence we can factor it as $|U\rangle = |S\rangle |E\rangle$. In this case, *S* and *E* can be regarded as “distinct” systems that each have “internal” states that are not directly involved in the interaction *H*_*SE*_. This condition of separability, or non-entanglement, assures only causal interaction between, and hence conditional statistical independence of, the components *S* and *E*.

The Hilbert-space decomposition $\mathcal{H}_U = \mathcal{H}_S \otimes \mathcal{H}_E$ induces a boundary $\mathscr{B}$ between *S* and *E*. Provided the joint state $|U\rangle$ is separable, i.e. $|U\rangle = |S\rangle |E\rangle$, this boundary $\mathscr{B}$ functions as a holographic screen separating *S* from *E*.^5^ A holographic screen is a boundary satisfying the holographic principle, a fundamental result in physics that asserts that a finite volume of state space or “bulk” cannot contain more externally-observable or accessible information than can be encoded on its boundary;^6^ it moreover asserts that if the system of interest is embedded in spacetime, the maximal information encodable on its boundary is $\mathcal{S}(\mathscr{B}) = A_{\mathscr{B}}/4$, where $A_{\mathscr{B}}$ is the area of $\mathscr{B}$ in Planck units (Almheiri *et al.*, 2021; Bousso, 2002; Fields *et al.*, 2022). As the interaction *H*_*SE*_ is defined at $\mathscr{B}$, we can in fact say precisely what this information is: at any time *t*, $\mathscr{B}$ encodes the eigenvalue of *H*_*SE*_ at *t* (Addazi *et al.*, 2021). We can, therefore, represent $\mathscr{B}$ as an array of *N* qubits (quantum bits), where *N* is the number of bits required to encode the largest eigenvalue, i.e. the maximum strength, of *H*_*SE*_. The boundary $\mathscr{B}$ therefore has an effective Hilbert space of dimension 2^*N*^. The interaction *H*_*SE*_ is “weak” in the required sense if $2^N \ll \mathrm{dim}(\mathcal{H}_S), \mathrm{dim}(\mathcal{H}_E)$, i.e. $\mathscr{B}$ being small compared to *S* or *E* corresponds to “sparse coupling” as required by the classical formulation of the FEP. Under these conditions, $\mathscr{B}$ functions as an MB between *S* and *E*.

Given the above characterization of $\mathscr{B}$, the interaction *H*_*SE*_ at a given time can be precisely specified as:


(1) $$ H_{SE} = \beta_k k_B T_k \sum_{i=1}^N M^k_i, $$

where *k* = *S* or *E*, the $M^k_i$ are single-qubit operators with eigenvalues +1 and −1, *k_B_* is Boltzmann’s constant, *T_k_* is temperature, and $\beta_k \geq \mathrm{ln}2$ is an inverse measure of thermodynamic efficiency. The coefficient $\beta_k k_B T_k$ is the energy per bit written and assures the compliance of *H*_*SE*_ with Landauer’s Principle (Landauer, 1961; Landauer, 1999). Informally, *H*_*SE*_ can be represented as a cycle in which *S* “writes” *N* bits on $\mathscr{B}$ that *E* then “reads,” after which *E* writes *N* bits that *S* reads in turn (Fields *et al.*, 2022; Fields and Glazebrook, 2022).

The variational free energy for either system can be defined as the difference between the bit string most recently written and the bit string that is subsequently read, i.e. the string most recently written onto the screen. The FEP requires any system to minimize the difference between expectation (the written string) and observation (the string subsequently read). As the limit of this process (in which writes and reads exactly match) corresponds to quantum entanglement, the FEP can be seen to be the classical limit of the principle of unitarity, i.e. the principle of conservation of information, upon which quantum theory is based; see Fields *et al.* (2022) for details and Fields *et al.* (2023) for a detailed comparison of the classical and quantum formulations of the FEP.

The classical formulation of the FEP starts with a random dynamical system; in this classical setting, separability between a system and its environment can be achieved by separating them in state space. We can then define the states of some thing, as against random fluctuations, by appealing to timescale separation: some states change slowly enough at some scale to be reliably re-identified as (effectively) the same states (Fields *et al.*, 2022); while other states change so quickly that they average out. Thus, the classical FEP is inherently multi-scale (Heins *et al.*, 2023; Ramstead *et al.*, 2021). The quantum information theoretic formulation does not assume a spacetime background; indeed, it is consistent with quantum-gravity models in which spacetime is both emergent from the underlying informational dynamics and system-relative (Fields *et al.*, 2024). It is, therefore, fully scale-free, applying in the same form to all systems; ranging from particle-particle interactions within the Standard Model, through the scales of molecules, cells, multi-cellular organisms, biological populations, and communities, through to the cosmological scale of quantum fields, black holes, and large-scale structures.

### Reference frames for perception and action

While the classical FEP abstracts the dynamics of *S* to a system-scale generative model, the quantum formulation abstracts *H_S_* to a collection of quantum reference frames (QRFs). As briefly introduced in (Fields *et al.*, 2021), a QRF is a physically implemented (hence quantum) measurement standard—meter sticks and clocks are canonical examples—that allows both observations and actions to be compared. They allow “differences that make a difference” (Bateson, 1972) between inputs to be detected by providing the fixed points against which differences are measured. Each QRF implemented by *S* can, therefore, be viewed as a generative model of some aspect or component of *E*’s actions on $\mathscr{B}$. The QRFs implemented by a system *S* collectively encode the complete set of inputs from *E* that can be processed and assigned actionable meanings by *S*.

Informally, QRFs can be regarded as encoding “categories” or “concepts,” including concepts specifying individual objects. Consider, as an example, walking into your office and seeing your laptop. Identifying your laptop as a specific object requires differentiating it from the background in which it is embedded, including the other objects in your office. This is done by noting certain distinguishing features of your laptop, e.g. size, shape, color, markings, etc.; these constitute the time-invariant “reference state” of your laptop. The network in your brain that implements these features and their expected values—hence, your generative model of your laptop—is the “reference” QRF for your laptop. The network that allows you to detect a specific, but non-identifying, state of interest of your laptop—a “pointer state” in physics language—such as a text or image displayed on the screen, is a “pointer” QRF specific to your laptop. The brain of a typical adult human clearly implements at least 100s of thousands of such QRFs.

**Figure 2.**
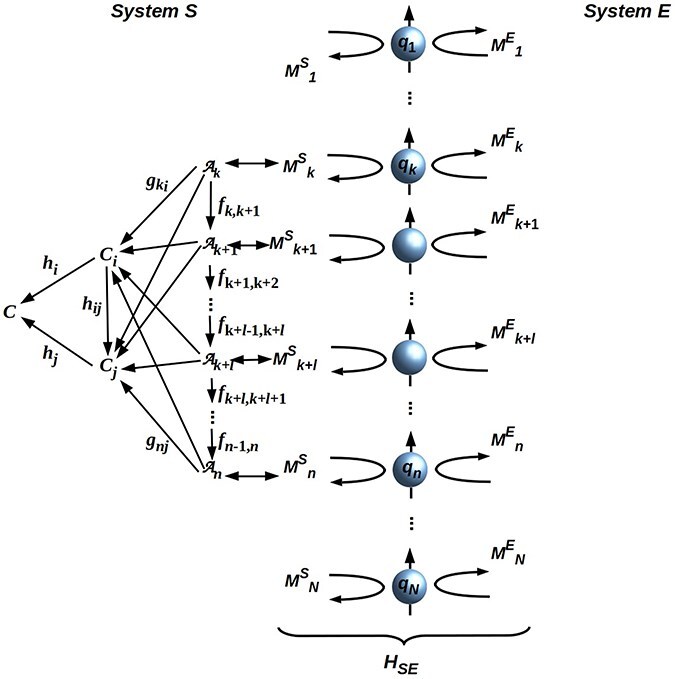
“Attaching” a QRF represented as a hierarchy of operators to an intersystem boundary $\mathscr{B}$ depicted as an ancillary array of qubits *q_i_*. Operators $M^k_i$, *k* = *S* or *E*, are single-bit components of the interaction Hamiltonian *H*_*SE*_. The node $\mathcal{C}$ is both the limit and the colimit of maps from and to the nodes $\mathcal{A}_i$; only leftward-going (cocone implementing) arrows are shown for simplicity. Adapted from Fields and Glazebrook (2020), CC-BY license.

Formally, a QRF implemented by *S* is a bidirectional hierarchy of operators that accepts input from, and transfers output to, a subset of the operators $M^S_i$ appearing in Eq. (1). These operators can be visualized as in Fig. 2. The system comprises a (nonunique) hierarchy of operators between a finite “base” set of operators $\mathcal{A}_i$ that interface directly to $\mathscr{B}$ and the (unique) operator $\mathcal{C}$ that is both the category-theoretic limit and colimit of maps to or from the $\mathcal{A}_i$, should such a $\mathcal{C}$ exist; see (Fields *et al.*, 2022; Fields *et al.*, 2023) for details and theoretical background. This representation is provably general for all repeatable, finite-resolution state measurements or manipulations (Fields *et al.*, 2022b).

As also shown in Fields *et al.* (2022b), any QRF can also be represented as a quantum operator $Q: \mathscr{B} \rightarrow \mathscr{B}$ that reads from and writes to some sector of $\mathscr{B}$. These operators may or may not commute, and hence may or may not be simultaneously deployable (Fields and Glazebrook, 2022). “Swapping” one QRF for another on the same sector of $\mathscr{B}$ is, effectively, changing the Hilbert-space basis used to define the $M^S_i$ acting on that sector of $\mathscr{B}$. Any system *S* that is separable from its environment *E* provably has “free choice” (i.e. not determined by its own past or that of *E*) of basis and hence of QRF for any sector of $\mathscr{B}$ (Fields *et al.*, 2022a); this free choice guarantees compliance with the Conway-Kochen theorems mentioned earlier.

As QRFs correspond to sensation-action loops *as mediated by their associated predictive-model components*, they encode expectations and enable detecting “differences that make a difference” to action; they encode semantics or “meaning” for *S* (Fields *et al.*, 2021). They are, however, also “just physics” as shown by their formal definitions as rehearsed above. This encoding of semantics by physics is fundamental to the notion of agency defined by the FEP, and in the quantum setting underlies the panpsychist notion that all distinct “things” are free agents that cannot be fully described by any locally-deterministic theory (Conway and Kochen, 2009).

### Space and time quantum reference frames

By adopting classical dynamical systems as a formal setting, the classical formulation of the FEP places agents in universal or clock time. The quantum formulation employs a notional “time” parameter *t* to define the propagator $\mathcal{P}_U(t)$, but this is not a measurable time. It moreover assumes nothing about an embedding space. Hence models of agents built within the FEP must specify whether, and how, each agent measures time and space.

As discussed in (Fields *et al.*, 2022), any agent *S* can be represented as implementing an agent-specific time QRF that counts actions on or writes to $\mathscr{B}$. The simplest such clock has no associated memory, so can only count to one. An agent equipped with this clock has only instantaneous experiences, none of which are remembered, although they may result, via the action of a learning algorithm, in changes to the internal dynamics *H_S_*. A finite memory for clock ticks is required for any ability to compare a current event—i.e. current sensory input—with one or more past events. Such comparisons clearly require, in addition to the clock-associated memory, a read/write memory on which (possibly highly coarse-grained) records of previous events can be written. This memory resource may be a sector of $\mathscr{B}$, in which case the memory is written on *E*, and so is stigmergic at the scale of *S*. It can, however, also be an internal, inter-component boundary sector, in which case the memory is stigmergic at the scale of the component that wrote it. Such internal memories are ubiquitous in biology, and are supported by multiple systems from the genome to the cell membrane at the cellular level and by local to large-scale cellular interactions in multicellular organisms. Both the short- and long-term memories for nutrient concentration levels that support chemotaxis in bacteria are well-understood, cellular-scale examples (Fields *et al.*, 2021).

While all vertebrates, all cephalopods, and at least most arthropods appear capable of spatial orientation, and hence must implement spatial QRFs, the prevalence of spatial QRFs in organisms in general is not at all clear. It is, moreover, not clear whether all organisms with spatial QRFs have three-dimensional (3D) spatial QRFs, or whether all organisms with even effective 3D spatial QRFs implement the kinds of agent-centered projective geometries that humans, and presumably all mammals and birds, appear to implement (Rudrauf *et al.*, 2017). Hence, while all but the most trivial agents implement multi-step clocks, agents may or may not implement spatial QRFs. Spatial QRFs clearly provide a format that is advantageous for cognition, because space provides a memory resource, a “place to put” information—e.g. an object or a stigmergic memory—where it can be kept distinct from other information and be retrieved later. Understood naively, this use of spatial location as memory location underlies many discussions of embodied, spatialized cognition, including spatialized imagination (Safron *et al.*, 2022), but is often left implicit. A full account of the use of spatial QRFs in general cognition, memory, and imagination must ground such spatialization in physics without assuming an objective spacetime embedding (i.e. without naively identifying an agent’s experienced spatially embedded objects with separable components of the agent’s external environment; cf. the concluding remarks in (Fields *et al.*, 2021).

### Thermodynamics and compartmentalization

As mentioned in connection with Eq. (1), acting on the world is energetically expensive. The fact that all agents must source thermodynamic free energy (TFE) from their environments is built into the FEP (Sengupta *et al.*, 2013), but is often left implicit in discussions of agents as inference systems. The requirement for sourcing TFE from *E* breaks an exchange symmetry on $\mathscr{B}$, dividing it into two distinct, non-overlapping sectors, a thermodynamic sector that manages TFE inputs and waste heat outputs—analogous to the power supply and cooling system of your laptop—and an informative sector that supports meaningful sensation and action (Fields *et al.*, 2022);^7^ see Fields *et al.* (2024) for details.

Living systems, unlike most artifacts (Ororbia and Friston, 2024), can be expected to operate on limited, if not severely restricted, TFE budgets. Living systems will, therefore, in general be forced to make tradeoffs that maximize the utility of the information that they expend energy to process and the actions that they expend energy to make. Keeping information processing and overt action—action on *E*—within the bounds of the TFE budget is the most basic task of an *attention system* (Fields *et al.*, 2021). The simplest mechanism of top-down attentional control is top-down control of the TFE supply—“turning off” QRFs that are not yielding useful information or productive action; in neurobiology, this could be read as sensory attenuation. As TFE resources can be expected to vary slowly compared to sensory inputs or actions, this top-down process can be expected to involve a system-scale metaprocessor, or “executive” control network that balances VFE reduction with TFE supplies (Kuchling *et al.*, 2022).

As mentioned above, QRFs that commute, and hence are simultaneously deployable, can be combined to form larger QRFs that recognize more complex events and enable more complex actions (see Friston *et al.*, 2024, for a classical treatment). Energetic constraints can, however, limit co-deployability, making in-principle commutative QRFs effectively noncommutative. Noncommuting QRFs can only exchange information causally, i.e. classically, so must be separated by boundaries. This requirement for classical internal communication compartmentalizes systems with limited TFE supplies, and by requiring internal classical actions—writing classical bits—increases their TFE requirements (Fields, 2024).

Any complete description of physical systems as uncertainty minimizers—hence any complete description employing the FEP—must include a description of how such systems minimize their uncertainties about both the availability and accessibility of TFE resources and their current and expected requirements for TFE. A requirement for productively allocating limited TFE resources will be better met by systems that incorporate feedback from their component QRFs that indicates their rate of TFE expenditure, and a self-monitor on the TFE distribution system that indicates current TFE availability and usage. These are the functions of the metabolic—or more generally, allostatic—stress signaling systems found in all organisms, including bacteria (Peters *et al.*, 2017; Fields *et al.*, 2024). At the whole-organism scale in mammals, these are brainstem functions that subserve arousal, including TFE distribution to the rest of the brain. The essential contribution of these systems to consciousness in humans and other vertebrates has been emphasized by Solms (2021) and Solms and Friston (2018).

The key role of TFE flows in experience rests on the distinction between informational content (on inner screens) and the regulation of how that content is read, which has no information content *per se*. The same distinction applies to the classical homologue of TFE flow; namely, the regulation of precision: in classical (i.e. Bayesian belief updating) formulations of the FEP, precision refers to the confidence afforded content; such that reading from the sensory sector of MBs depends upon the precision afforded content. This distinction is sometimes cast in terms of first and second order statistics, respectively (Feldman and Friston, 2010). As intimated above, there is an interesting link between quantum and classical formulations of the ensuing metacognitive architectures. For example, gauge theoretic treatments of the FEP (Sengupta *et al.*, 2016) speak to precision as the Fisher information metric of statistical manifolds upon which belief updating unfolds. On this view, the thermodynamic cost of belief updating (i.e. movement on a statistical manifold) corresponds to the information distance between consecutive updates, which increases with precision and, via the Jarzynski equality (Evans, 2003; Jarzynski, 1997; Seifert, 2005), the requisite thermodynamic work. This speaks to an intimate relationship between TFE flow, precision, attention and experience.

## A “simple” agent with only non-imaginative experience

**Figure 3.**
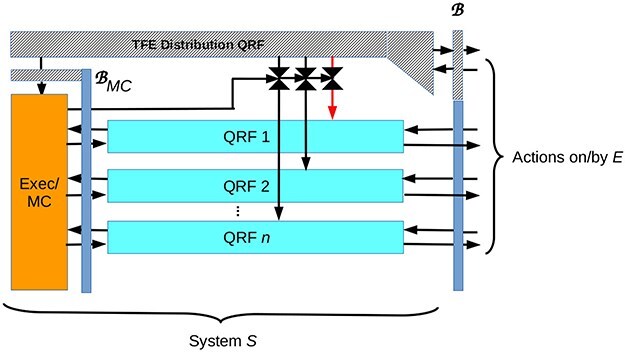
A minimum architecture for an agent with multiple QRFs (light blue rectangles) and attentional control via TFE resource distribution implemented by an executive/metacognitive system (orange rectangle). Both the agent *S* as a whole and the executive/metacognitive component have boundaries/MBs; the MB of the executive/metacognitive component is an “inner screen.” The thermodynamic sector of each boundary/MB, as well as the TFE distribution system are shown by grey hatching; the informative sectors of boundaries are dull blue. Red vertical arrow indicates that TFE inputs to some QRFs, e.g. homeostatic interoception, can over-ride attenuation by the executive/metacognitive system. Equating what is experienced with what is encoded on the informative sector of the boundary/MB, the overall agent *S* experiences sensations from and actions on its environment *E*, while its executive/metacognitive component experiences inputs from and outputs to the QRFs labeled 1, 2, $\dots \ n$. Neither *S* nor its executive/metacognitive component have imaginative experiences, i.e. experiences of something other than their interactions with their own environments.

With these preliminaries, we can construct a minimal architecture for an agent that experiences sensations from—and actions on—its environment *E*, but does not experience imaginations. This architecture is illustrated in Fig. 3. This agent implements multiple QRFs that read from and write on the informative sector of its boundary or MB $\mathscr{B}$. These QRFs implement the agent’s processing and categorization of sensations and its execution of actions; hence they collectively specify the agent’s repertoire of sensory and action capabilities. Attentional control is implemented by an executive system, which is metacognitive in the sense that it both monitors and exerts control over the behavior of the “lower-level” QRFs that act directly on $\mathscr{B}$. In particular, the executive/metacognitive system allocates TFE to each QRF to control its processing ability and/or rate (in classical formulations this “rate” can be read as the precision or attention afforded to the sensory sectors of MBs). This executive/metacognitive system is itself an agent, with its own boundary/MB and its own environment, the local components of which are the QRFs implemented by *S*. Like any FEP agent, the executive/metacognitive system senses and acts on its environment via its own, meta-level QRFs, which collectively implement its meta-level predictive model.

We assume that each of the low-level QRFs implemented by the system incorporates a TFE usage monitor as discussed above, and that the executive/metacognitive system allocates TFE to these QRFs in accord with their usage, their current priority as computed by the executive/metacognitive system, and the overall availability of TFE. Under conditions of insufficient TFE, the executive/metacognitive system can implement a “stress response” that may include differential activation of QRFs or simply shutting down some processes altogether. Some QRFs, for example QRFs that monitor and control resources essential for homeostasis, may be refractory to TFE regulation by the executive/metacognitive system. If an induced stress response produces detectable actions on $\mathscr{B}$, e.g. molecular secretions, bioelectric field changes, bodily movements or vocalization, the system *S* will experience its stress response; if no detectable actions on $\mathscr{B}$ are generated, it will not. If the TFE supply falls below the threshold required to operate its QRFs, *S* will cease to have experiences.

The internal boundary $\mathscr{B}_{MC}$ of the executive/metacognitive system is an “inner screen” that mediates classical communications between the executive/metacognitive system and the QRFs that it controls. A system with this architecture may have many other internal boundaries and hence many other inner screens; for example, any or all of its QRFs may have internal boundaries between hierarchical processing layers. This potential complexity is elided in Fig. 3 and as will be seen below, is irrelevant to what *S* experiences.

If we equate the experience of a system with the sensations from its environment that are encoded by its boundary/MB, then *S* experiences inputs from and, via their effects on subsequent sensations, outputs to its environment *E*.^8^ The executive/metacognitive component of *S* experiences inputs from and outputs to the QRFs implemented by *S*. These experiences are correlated—*S*’s executive/metacognitive component only experiences a QRF output when *S* experiences an input to that QRF—but have different content; they are, in particular, experiences of entirely different environments. If we take *S* to represent an *E. coli*, for example, *S* experiences some external milieu, e.g. the nutritious medium in a laboratory culture dish, while its internal executive/metacognitive component, a complex of gene regulatory pathways, experiences the macromolecular milieu inside the *E. coli* cell. Should any of the components of *S*, e.g. *S*’s QRFs, have internal boundaries, these internal boundaries will encode experiences for the internal components that they bound, but unless some mechanism also encodes them on the boundary of *S*, they will be irrelevant to the experience of *S*. The architecture shown in Fig. 3, therefore, cleanly separates the experience of *S* from the potential experiences of any of its components without having to invoke an *a priori* restriction on the experiences of components, e.g. the “Principle of Exclusivity” employed in IIT 3.0 (Tononi, 2015).

While an agent such as that shown in Fig. 3 has no imaginative experience, it may be cognitively sophisticated, e.g. having internally-encoded memories and the ability to compute EFEs and hence to choose alternative action policies. To continue the previous example, *E. coli* cells are able to choose between and execute different behavioral policies subserving chemotaxis. To do this, they must encode internal memories and compute EFEs, which they do via well-characterized biochemical networks (Fields *et al.*, 2021). Organisms much more complex than bacteria, e.g. “lower” vertebrates such as fish or reptiles, are clearly capable of context-dependent action selection without any evidence of imaginative abilities. When processes in such systems are “off line”—decoupled from the environment—they may be entirely non-conscious.

While a Fig. 3 agent has no imaginative experiences, it can monitor its own cognition, including its metacognitive computations, provided these processes couple to the environment by generating actions on $\mathscr{B}$ as output. Systems without imagination are not, therefore, necessarily metacognitive zombies. For example, a Fig. 3 agent could engage in conscious, deliberate planning by reporting metacognitively-executed planning steps to itself via vocalized speech. It could, if equipped with the appropriate QRFs, use pencil and paper to record notes on its deliberations, draw diagrams, or perform calculations. However, it could only do these things overtly, and hence in a sense publically. Lacking imagination, it could not engage in inner speech, inner imagination, or computation “in its head.” It would, in other words, be incapable of any *experienced* covert actions.

## Equipping agents with imaginative experience

### Why is imaginative experience problematic?

Imaginative experience raises three questions for the FEP. Employing the idea of an “inner screen” $\mathscr{B}_{in}$ on which such experiences are encoded, they can be posed as:

1. How can QRFs defined on $\mathscr{B}$ be re-deployed to access information encoded on an inner screen $\mathscr{B}_{in}$?
2. How are the contents of imaginative experiences generated and written onto $\mathscr{B}_{in}$?
3. How can the contents of imaginative experiences be surprising?

Answering these question requires an architectural model of an agent *S* that:

- Specifies the architectural and functional relationship between the *S*-*E* boundary $\mathscr{B}$ and the inner screen $\mathscr{B}_{in}$.
- Specifies the control structure that switches attention between inputs encoded on and outputs directed to $\mathscr{B}$ and inputs encoded on and outputs directed to $\mathscr{B}_{in}$.
- Explains how $\mathscr{B}_{in}$-directed actions are initiated and executed.
- Explains how $\mathscr{B}_{in}$-directed actions can be unpredictable by, and hence surprising to *S*.

To construct such a model, we will employ two assumptions. First, we assume that the same set of QRFs that are used to interpret input from and act on the external environment are also used to implement imaginative experience by interacting with $\mathscr{B}_{in}$. This is a strong constraint, as it restricts *S*’s repertoire of imaginative experiences to a subset, not necessarily proper, of its repertoire of environmentally-driven experiences. It also implies that $\mathscr{B}_{in}$ requires no distinct thermodynamic sector, i.e. the thermodynamic requirements of imaginative experience are handled by the same mechanisms that handle the thermodynamic requirements of non-imaginative experience, with TFE both sourced and exhausted through the thermodynamic sector of $\mathscr{B}$. Second, we assume that while imaginative experience may be coarse-grained relative to externally-sourced experience, it is not required to be so coarse-grained. Hence we assume that all QRFs capable of contributing to imaginative experience do so at full resolution.

### Sector-specific gating between $\mathscr{B}$ and $\mathscr{B}_{in}$

**Figure 4.**
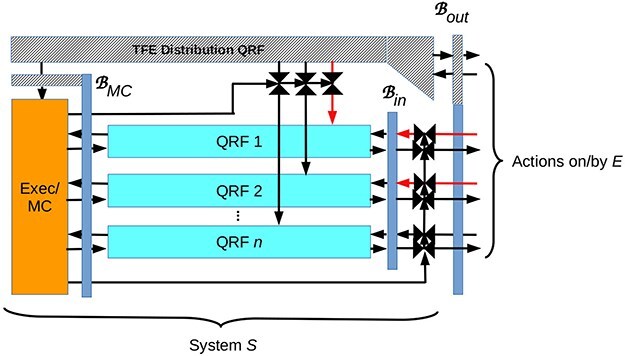
Adding an inner screen $\mathscr{B}_{in}$ and a control layer to the architecture shown in Fig. 3 enables imaginative experiences that employ the same QRFs as non-imaginative experience. The agent *S* can, with this architecture, choose to perceive and act on the external environment *E* or on an “imagined environment” encoded only on $\mathscr{B}_{in}$. Horizontal red arrows indicate that some external inputs can over-ride being attenuated by the executive/metacognitive system; this over-ride capability can be expected to be both state- and trait-variable.

The simplest solution to the problems posed above is an architecture in which *S*’s QRFs act *only* on, and hence *S* experiences *only* sensations and actions encoded on, an inner screen $\mathscr{B}_{in}$. A minimal architecture implementing this solution is shown in Fig. 4. This architecture modifies that of Fig. 3 by interposing an inner screen $\mathscr{B}_{in}$ between *S*’s QRFs and the informative sector of its boundary—relabeled ‘$\mathscr{B}_{out}$’—with *E*. It also adds a control layer of QRF-specific gates that allow input and output to flow between $\mathscr{B}_{in}$ and $\mathscr{B}_{out}$. If these gates are all open, $\mathscr{B}_{in}$ is rendered “transparent” and *S* experiences sensations from and acts on *E*, i.e. the architecture functions exactly like the architecture of Fig. 3. If the gates are closed, *S* experiences only sensations that result from its own actions on $\mathscr{B}_{in}$. Some inputs, e.g. homeostatic interoception, may be refractory to executive control, and hence difficult or even impossible to turn off. If some gates are open and some are closed, *S* experiences a combination of external and imaginative content.

Individually gating input and output channels from *S*’s QRFs, and hence sectors of both $\mathscr{B}_{in}$ and $\mathscr{B}_{out}$, enables *S* to experience imaginations in some modalities and external inputs in others. Humans clearly have this ability, e.g. the ability to engage in unvoiced inner speech while driving a car. At a higher level of resolution, it enables a mix of imaginative and external inputs in a single modality, e.g. hallucinating an imaginary person in an externally perceived room. It is worth noting that cross-modality mixtures of imagination and externally-driven experience are typically both voluntary and useful, while within-modality mixtures are sometimes involuntary and pathological.

Externally-imposed selective pressures on living systems can be expected to yield control gates between $\mathscr{B}_{in}$ and $\mathscr{B}_{out}$ that do not completely close, and can be over-ridden by external signals of sufficient amplitude. Selective pressures on mammals, for example, can be expected to yield ventral attention systems that can override the dorsal attention system. In humans, this ventral override capability can be defeated pharmacologically, e.g. by alcohol. “Leaky” valves between $\mathscr{B}_{in}$ and $\mathscr{B}_{out}$ can be expected, in general, to produce a somewhat fuzzy and maleable boundary between imagined and external worlds, an effect that is evident in hyperphantasia (Zeman, 2024), schizotypy (Humpston *et al.*, 2017), psychosis, and intoxication (Carhart-Harris and Friston, 2019).

### Generating imaginative contents

In the architecture of Fig 4, imaginative contents are actions on $\mathscr{B}_{in}$ that are fed back into the QRFs that generated them instead of being passed outward to $\mathscr{B}_{out}$. They are, therefore, generated in exactly the same way that actions on the external world are generated. There is, in other words, nothing metaphysically “special” about the generation of imaginative content.

Whether *S* is a metacognitive zombie similarly depends on the QRFs it implements just as it does in the imagination-incapable architecture of Fig. 3. As in Fig. 3, *S* does not experience what is written on the executive/metacognitive system’s boundary $\mathscr{B}_{MC}$. Only the executive/metacognitive system experiences these data. Unlike in Fig. 3, an imagination-capable *S* does experience the results of writing selected covert actions on $\mathscr{B}_{in}$, e.g. inner speech, visual images, or other modal imaginings. Because the processing capabilities of the QRFs do not differ between Figs 3 and 4, nothing can be imagined in Fig. 4 that cannot be experienced overtly by either architecture. Nothing can be said, for example, with inner speech that cannot also be voiced, though the consequences of voicing thoughts to *E* may be very different from the consequences of keeping them covert. The architecture of Fig. 4 is, therefore, fully consistent with the “flat mind” proposed on the basis of both behavioral and neurocognitive data by Chater (2019).

### How can imaginative content be surprising?

We are now in a position to address the primary challenge to theories of imagination posed by the FEP: if imaginations are internally generated, how can they be surprising? Imagination would be useless if its content was not surprising. What good would inner speech be, for example, if you could not discover anything by using it? If an agent were fully aware of its generative model, consciously worked through all of its predictions, and could consciously monitor its actions, it could never be surprised by its covert actions on its own inner screen. Such a system could learn nothing from imagination; an imagination capability, and hence an inner screen, would just be costly architectural baggage.

In the architecture of Fig. 4, covert action generates a prediction for the next imaginative input. This prediction process has no access to the immediately-preceding action selection, which is performed by the executive/metacognitive system. What does this imply about how the executive/metacognitive system reads and writes from its boundary? The environment of the executive/metacognitive system is (mostly) the collection of QRFs. Their behavior—what they report as output to the MC—is what triggers the surprise. The executive/metacognitive system has a model of its environment—a model of what the QRFs do and how they work—but this model must be learned and so cannot be fully accurate. This reflects the fact that the executive/metacognitive system is just an ordinary agent living in an ordinary environment, even though this environment is locally internal to *S*.

A system *S* with the architecture of Fig. 4 can be surprised by its imaginative experience, therefore, because it *is not aware* of its generative model and *does not know* how its own actions are generated. We can say this as: we (systems like *S*) do not know how our QRFs work, and neither do our executive/metacognitive systems. The Executive Network is controlling a system that it only has a coarse-grained, heuristic model of. This idea that systems are ignorant of their own internal processing is, again, fully compliant with the “flat mind” concept, and with views in the philosophy of mind that treat self-knowledge and knowledge of other minds alike as based on inference (Sellars, 1997). It follows, moreover, as a constraint on all classical information processors via Ashby’s Law of Requisite Variety (Ashby, 1956), and as a theorem for any bounded quantum system (Fields *et al.*, 2024).

### Metacognition as an expected free energy calculator

The FEP models planning as policy selection following computation of expected free energies for possible courses of action. A primary task of the executive/metacognitive system is, therefore, EFE calculation. As we have seen, however, the executive/metacognitive system cannot have a fully-accurate model of the QRFs it interacts with, so it cannot have a fully-accurate model of either the sources of its inputs or the capabilities of its outputs. It cannot, therefore, reliably compute EFEs.

Systems with imaginative capability have, therefore, a qualitative advantage over systems that lack this ability: a Fig. 4 system can supplement is unreliable models of how its QRFs work by covert experimentation. This process is still unreliable, as the results of such experiments must still be interpreted using an unreliable model, but experimentation can be expected to improve reliability *provided the results can be surprising*. The executive/metacognitive system is, in other words, precisely in the position of an empirical scientist, as indeed all agents are.

The advantage of experimentation via imagined, covert action over straight prediction using an unreliable model of QRF behavior can be expected to increase as the planning depth, and hence the anterograde extent of the “cognitive light cone” (CLC) (Levin, 2019) of *S* increases. It can also be expected to increase as the spatial extent of the CLC increases, as how spatial and object QRFs relate may be difficult to compute from their executive/metacognitive system-resident models. Systems that have highly practiced overt actions, however, may not require imaginative simulation to adequately plan instances of similar actions, particularly if they can experience relevant real-time sensory feedback from *E* while carrying the action out.

## Discussion

### The implementation of imagination

The architectures depicted in Figures 3 and 4 are intentionally generic, implying no specifics about implementation beyond compartmentalization into the indicated components. In an artificial system, these boundaries could be realized through classical application programming interfaces (APIs) with any underlying classical data structures. The processing components could, in principle, be implemented by computational modules with any sufficiently capable architecture—from a finite lookup table or production system to a fully-unitary quantum process. Aside from differences in running time and TFE consumption, the behaviors of these alternative implementations could be made identical.

It is worth emphasizing the implementation independence of the current, FEP-based approach in contrast to IIT 3.0. Within the FEP framework, implementation independence is principled: observations of a system cannot penetrate beyond its MB and hence cannot reveal implementation details. This is, obviously, a black-box assumption, and the requirement of implementation independence can be traced to the fundamental theorems of Moore (1956), and beyond them to the Church-Turing thesis. Experimental disassembly accompanied by theoretical decomposition is clearly allowed within the FEP framework, with the property of implementation independence then transferred to the components. These distinctions reflect the fact that the FEP framework is fully scale-free, while IIT 3.0 assumes a particular scale that is regarded as “of interest” for the analysis of some computational outcome.

Switching from a generic perspective to that of modeling specific cognitive systems, e.g. humans, it is necessary first to say how the informational boundaries specified in Figs. 3 and 4 relate to functional or anatomical boundaries in the implementing system. It is natural to take the system *S* to be implemented, in humans or other animals, by the nervous system, including both central (CNS) and peripheral (PNS) components, and the environment *E* to comprise all other components of the organism’s body as well as the external environment. This way of relating informational and anatomical boundaries makes the nervous system the “agent of interest” and focuses attention on how it observes and acts on the rest of the body as its immediate environment. This mapping allows us to make sense, in particular, of the rest of the body as the exclusive source of TFE for the nervous system, and of homeostatic interoception and its associated control functions as observation and (partial) control over the functioning of the rest of the body.

This way of relating the boundaries clearly does not work in the case of non-neural organisms. As there is currently no evidence for imaginative cognition in non-neural organisms, this does not pose a problem for the current analysis. However, there are also considerations that tell against the idea that the boundary of *S* (the subject of typical whole-system level conscious experience in human-like organisms) extends to the peripheral nervous system. It is common, for example, to experience features across different sensory modalities co-located in the same 3D space (e.g. the sound and visual appearance of my fingers snapping). It is difficult to account for this sort of “unity” of conscious experience if $\mathscr{B}_{in}$ resides very close to the sensorimotor periphery, where cross-modal information has not yet been integrated into a shared spatial reference frame, and visual and auditory information would presumably be written to and read from distinct “locations” on the peripheral screen. Classic arguments for Cartesian intuitions, such as the reality of “phantom limb” syndrome, also tell against the inclusion of peripheral systems as necessary components of *S*, since qualitatively similar experiences may occur in the absence of the peripheral parts.^9^

While the boundaries of *S* may thus remain inevitably fuzzy at the population level, it is easier to generalize about the core. In our model, the fundamental enabler of both experience and information processing is TFE flow. The most basic function of awareness is, therefore, the monitoring and control of TFE flow. In mammals, this is a function of the PNS together with brainstem and mid-brain nuclei. Hence our model is broadly consistent with that of Solms (2013), which emphasizes the importance of interoception—the sensing of internal bodily states—in generating consciousness. Solms proposes, in particular, that the Freudian id, associated with instinctual drives and emotions, is a necessary enabler of consciousness; see Safron (2021b) for critical commentary. We would phrase this differently, as the claim that the experience of homeostatic interoception is the most fundamental kind of experience for a neural organism. In either case, interoceptive processes form the foundation of conscious experience, upon which higher-order cognitive processes are built. The idea of interoception as observation of the body clearly aligns with our approach, highlighting that what a system *can be* conscious of is encoded peripherally, while *whether* the system is conscious of those contents is encoded centrally by the energy/arousal distribution system, the executive system, and their interaction. We can expect highly distributed neural correlates of consciousness, and look forward to the Global Neuronal Workspace versus IIT experiments (Consortium, 2023) to test this expectation.

### Memory and the phenomenology of imagination

The processes of imagination and memory are inherently influenced by our internal bodily states. Arousal and interoception shape our immediate conscious experience, both via attentional control and affect, and influence how we reconstruct past events. Through the integration of interoceptive signals with memory, the metacognitive system generates rich, embodied simulations. Imaginatively reconstructed episodic memories are thus connected to our internal physiological states.

Imaginatively reconstructed episodic memory serves multiple functions, primarily to update our predictive models of the world (Friston, 2017). Retrograde mental time travel (remembering the past) often serves anterograde mental time travel (planning for the future), as the metacognitive system gets refined based on past experiences. Episodic recall is not exclusively future-oriented; it also reinforces self-identity and supports emotional regulation.

The subjective-timescale model presented by Zakharov *et al.* (2021) shows that by collecting episodic memories based on salient events, an agent has flexible temporal reasoning that can support both retrospective analysis and prospective planning. The Husserlian concept of a “living present” that integrates past, present, and future experiences (Albarracin *et al.*, 2022; Bogotá and Djebbara, 2023) depends on tight coupling between retrospective and prospective processes. The agent can simulate both past and future scenarios, supporting counterfactual thinking and enabling individuals to learn from past experiences and imagine potential future outcomes (Parr and Pezzulo, 2021). The hierarchical structure of these models, with multiple timescales of representation, mirrors the brain’s ability to integrate immediate experiences with longer-term memories and plans.

Covert actions are written on the inner screen $\mathscr{B}_{in}$ to simulate past experiences or imagine future scenarios without engaging in overt behavior. Thus, episodic memory serves multiple functions, including updating predictive models and supporting both retrograde and anterograde mental time travel (Friston, 2017). The surprise generated by covert actions on the inner screen, due to the metacognitive system’s imperfect model of the QRFs, can lead to new insights or unexpected associations. This surprise-driven process could account for the creative and adaptive aspects of episodic memory, enabling the agent to discover novel connections between past experiences and potential future situations.

Constant, Friston and Clark (2024) define creativity as a rolling process of hypothesizing solutions to problems, testing them, and evincing solutions that are both novel (statistically different from other products) and apt (responding to task demands). In their simulations, creativity emerged when agents were placed in “exploration bubbles” that perturbed their normal operating conditions. A creative process could be implemented through the interaction between the metacognitive system and the QRFs. When faced with a novel problem, the metacognitive system could initiate a process of “cognitive husbandry” by manipulating the inner screen $\mathscr{B}_{in}$ to create challenging scenarios. The QRFs, representing past experiences and potential future states, would then interact with these perturbed scenarios on $\mathscr{B}_{in}$, potentially leading to novel combinations and insights.

The surprise generated by these novel combinations manifests as unexpected patterns of activation on $\mathscr{B}_{MC}$. Such surprising patterns could then feed back to the metacognitive system, potentially leading to updates in the agent’s world model and decision-making processes. Creativity thus takes place within the constraints of the agent’s existing model space. Constant, Friston and Clark (2024) suggests that this addresses the Enlightened Room Problem (i.e. the problem of accounting for how prediction-error-minimizing agents can seek novelty and act creatively) by showing how novel solutions can emerge without expanding the bounds of the agent’s prediction arena.

The separation between the metacognitive system and the QRFs explains why episodic recall can feel like “re-experiencing” rather than simply retrieving information, as the process involves actively reconstructing experiences on the inner screen rather than accessing stored data.

### Pathologies of the inner screen

The model of imaginative experience presented here suggests that disturbances in mental functioning, such as depression and anxiety disorders, may at least in some cases result from disruptions in the functioning of the inner screen $\mathscr{B}_{in}$ and/or its relationship with the external world via $\mathscr{B}_{out}$. At the core of many of these disorders are disruptions of allostasis and interoception (Friston, 2023), which may manifest as alterations in the content and dynamics of the inner screen. We do not, of course, claim that the remarks below constitute a full account of either the etiology or the phenomenology of such conditions.

In depression, the brain’s internal model, whose traces appear on $\mathscr{B}_{in}$, eventually becomes heavily biased toward negative predictions and inefficient energy regulation. This aligns with Barrett *et al.*’s view of depression as a disorder of allostasis (Barrett *et al.*, 2016). Persistently pessimistic interoceptive predictions may lead to the subjective experience of fatigue, low mood, and somatic symptoms characteristic of depression. While negative imagery is an important feature of clinical depression, the inability of internal components to take covert actions that produce surprising effects on the inner screen—whether positive or negative—could lead to stable imaginative experience with negative valence, and hence is arguably sufficient to bootstrap the disorder. Since action on the internal screen is, as discussed above, no different in kind from the initiation of external action, depressed individuals can be expected to be less likely to visit novel paths in the external world (i.e. they lack motivation to pursue changes from the status quo), and thus come to predict less variety, a vicious cycle.

Moreover, chronically accurate prediction of sensory inputs (an internal analogue of the “depressive realism” hypothesis (Moore and Fresco, 2012)), which is facilitated by a reduced variety of experiences, may contribute to literally low-energy (i.e. low-TFE) states of the internal components responsible for initiating covert action: only unexpected inputs require posterior belief updates, necessitating computation that would attract metabolic resources. Lack of belief updating may then over time give rise to a lethargic condition in the mechanisms responsible for initiating covert action, a mechanism that might also partially explain core aspects of aging and senescence. Energetic inefficiency, as proposed by Barrett *et al.* (2016), manifests as psychomotor retardation, lack of motivation, and the cognitive difficulties often seen in depression, as the system struggles to allocate resources for generating varied and dynamic content on the inner screen.

A key feature of both depression and anxiety disorders is ruminative episodic memory. In our model, rumination is a maladaptive attempt to update internal models, where the metacognitive system becomes stuck in a loop of recalling negative experiences (Jin *et al.*, 2024). This process occurs on the inner screen, with the system repeatedly replaying negative scenarios without successfully integrating this information to improve future predictions (Knolle *et al.*, 2023). This “stuck” state might result from overly precise priors about negative outcomes or an imbalance in the gating between $\mathscr{B}_{in}$ and $\mathscr{B}_{out}$, leading to a stable attentional focus on negatively valenced imaginative content—e.g. ruminative narrative—with reduced attention to external sensory input.

The gating mechanism in depression may strongly favor $\mathscr{B}_{in}$ over $\mathscr{B}_{out}$, explaining the tendency for depressed individuals to become absorbed in negative internal narratives at the expense of engaging with the external world. In anxiety disorders, this manifests as excessive simulation of negative future scenarios on the inner screen (Limongi *et al.*, 2023). Depression might involve a reduction in the counterfactual depth of the inner screen’s content (Rappe and Wilkinson, 2023). The ability to generate or utilize alternative scenarios, particularly positive ones, may be impaired. This could account for the difficulty depressed individuals have in imagining positive future outcomes or alternative interpretations of events, all of which would normally be simulated on the inner screen. The metacognitive layer manages the inner screen’s content and may become locked into a state where it continually reinforces negative models. This explains the persistence of depressive symptoms despite environmental changes, as the inner screen remains dominated by negative content even when external circumstances improve.

Depression thus entails altered precision weighting, where inappropriately high precision is assigned to negative content on the inner screen, with low precision given to positive sensory inputs from $\mathscr{B}_{out}$. This explains why depressed individuals often discount positive experiences and overemphasize negative ones—the inner screen becomes “stuck” displaying high-precision negative content. These factors interact in a self-reinforcing cycle on the inner screen. Altered interoceptive predictions lead to negative bodily sensations being prominently displayed, reinforcing the gating bias toward internal states and further reducing engagement with potentially positive external inputs. The impaired counterfactual thinking limits the ability to populate the inner screen with alternative scenarios or solutions, maintaining the pessimistic internal model.

We can also account for unusual imaginative experiences, such as phantom limb pain (Feldman, 2016) or hallucinations in psychosis (Knolle *et al.*, 2023). In the case of phantom limbs, individuals continue to experience sensations, including pain, in a limb that has been amputated. The persistence of the brain’s internal model or “body schema” of the missing limb (Makin *et al.*, 2013) accounts for this. The mismatch between the brain’s expectations (the presence of the limb) and the actual sensory input (absence of the limb) leads to the generation of phantom sensations. These experiences, along with hallucinations in psychosis, can be understood as mismatches between interoceptive predictions and actual sensory input (Barrett and Simmons, 2015; Seth, 2013; Seth *et al.*, 2012). The brain, attempting to minimize prediction error, generates phantom sensations or hallucinations to reconcile this discrepancy. In schizophrenia, this mismatch might be exacerbated by altered precision-weighting of internal versus external signals (Limongi *et al.*, 2023; Friston, 2023).

In some cases, psychosis might be better understood as a difficulty in distinguishing or appropriately using counterfactual versus factual hypotheses (Rappe and Wilkinson, 2023). This could manifest in several ways. There might be a “reality monitoring” breakdown as described by Simons, Garrison and Johnson (2019), where counterfactual parts of the model are misidentified as pertaining to the actual world; or there could be a loss of access to parts of the counterfactual model, resulting in an inability to access certain alternative hypotheses. Poor counterfactual underpinning might occur, in which the agent lacks the ability to generate sufficient alternative hypotheses. There could also be problems with subjective markers of reality, leading to difficulties in assigning appropriate “reality tags” to experiences (Rappe and Wilkinson, 2023). The internal and external points of reference, and hence QRFs, used by humans to distinguish “real” from “counterfactual” information are poorly understood and may vary widely between individuals, and studies of self-deception (von Hippel and Trivers, 2011) and motivated reasoning (Rigoli *et al.*, 2021) demonstrate. Further work in this area is clearly needed.

Imaginative experiences as internal simulations gated from external input can account for both normal imaginative functions and their alterations in various psychopathological conditions. The same underlying mechanisms can lead to adaptive simulations that guide behavior or maladaptive patterns that maintain disorders. In schizophrenia and psychosis, we can explain how disruptions in the balance between internal and external inputs can lead to reality distortions and cognitive disorganization (Sterzer *et al.*, 2018; Limongi *et al.*, 2023). Generating “inaccurate” or counterfactual hypotheses is not unique to psychosis but is a crucial part of normal cognition (Rappe and Wilkinson, 2023). The neurotypical brain constantly generates counterfactual, de-coupled hypotheses as part of its rich tapestry of cognitive processes. This “counterfactual depth” underlies our ability to engage in complex reasoning, planning, and even our sense of reality itself (Seth, 2014; Wilkinson, 2021).

### The phylogeny of imagination

The meaning, and therefore the phylogenetic distribution, of “cognition” is a subject of active debate (Bayne *et al.*, 2019), with positions ranging from the pancognitivism of Maturana and Varela (1980) and many others in the basal-cognition movement to a primary focus on apparently-unique features of human cognition (Dehaene *et al.*, 2022; Penn *et al.*, 2008). It is not, however, clear where or how often in phylogeny a transition from Fig. 3 to Fig. 4 architectures has occurred. While imagination may be most associated, functionally, with “thinking” and introspection, it also underlies such activities as dreaming and imaginative play. Organisms that entirely lack the kind of symbolic reasoning emphasized by Penn *et al.* (2008) or Dehaene *et al.* (2022) may nonetheless experience *some* internally-generated content in *some* circumstances. Whether non-human mammals and other big-brained organisms dream is an active research question (Malinowski *et al.*, 2021; Manger and Siegel, 2019); a similar question could be asked about imaginative play.

We can also ask this phylogenetic question from an evolutionary perspective: In what organisms would a capacity for covert action be selectively advantageous? Many social situations call for covert action in the form of unexpressed thoughts or emotions, and selection for social adeptness was (and still is) strong in the human lineage (Dunbar and Shultz, 2007). While the ability to deceive is enhanced by the ability to self-deceive (von Hippel and Trivers, 2011), the development of robust self-deception in a system incapable of covert action seems unlikely. The distinction between behaving in a way that is effectively deceptive, which may characterize even individual cells in a multicellular organism (Fields and Levin, 2020a), and intentionally deceiving via covert action is, however, difficult to draw in the absence of independent evidence of covert experience. Commonplace instances of mimicry, for example, are deceptive but do not suggest any experience of active deception.

We are left, therefore, with the usual conundrum about any form of experience, one that only advances in comparative functional neuroscience is likely to resolve. Covert action *could have* evolved early and often, and would have been selectively advantageous in many contexts. It could, however, also have evolved late and seldom—and possibly only in the human lineage—with the evident abilities of other systems to deceive being explicable without appeal to covert action. The enormous diversity in human imaginative abilities, most of which remains uncharacterized by functional neuroscience, suggests that the latter option is at least plausible.

### Human variation in state and trait imagination

Individual humans are capable of—and when it is involuntary, suffer from—state variability in imagination, and human populations exhibit substantial trait variability in imagination. That behaviors involving high levels of automaticity—including “flow” behaviors exhibiting high-level expertise—involve little to no introspective cognition or imaginative self-awareness and are characterized by strongly-attenuated default network activity is well-established. In the notation of Fig. 4, these states involve “turning off” covert action by “turning on” the flow of action to the external environment. Such states are surprisingly common, including all of “type 1” cognition (Evans and Stanovich, 2013) as well as everyday activities such as grammatical (native) language use. State variability in covert experience can also manifest as pathologically uncontrollable imaginative experience, e.g. in depression or psychosis. State variability of this kind has been described in terms of Bayesian precision modulation (Friston, 2023); Fig. 4 can be seen as assigning a locus to the experiential effects of such modulation.

More significant from the present perspective is the large trait variability in human imaginative capabilities. Heavey and Hurlburt (2008) showed by random experience sampling that some behaviorally typical, apparently unimpaired, and undistressed humans experience essentially no inner speech or essentially no visual imagination. Palombo *et al.* (2015) found that some healthy adults experienced essentially no imaginatively-vivid autobiographical memories. It is now known that some people experience little or no imaginative content in some or all modalities (Zeman, 2024). This condition of aphantasia is not a clinical syndrome and those who experience it typically have no obvious deficits,^10^ while the opposite condition, hyperphantasia, can present as schizotypy or symptomatic schizophrenia, as noted above.

The fact that individual humans can be behaviorally typical, apparently unimpaired, and undistressed while experiencing essentially no “inner life” is counter-intuitive for many, and saddles any theories of consciousness that build in requirements for covert inner experience with the consequence that some behaviorally typical, unimpaired and undistressed humans are in fact philosophical “zombies.” From the present perspective, it merely suggests that some people have cognitive architectures much more like Fig. 3 than like Fig. 4, and that most of us cannot tell the difference by external observation.

## Conclusion

We have showed here how to implement a system that can surprise itself with its imaginings, something that humans are clearly capable of doing. We have used only minimal assumptions that are compliant with the FEP; hence the model is completely generic and could apply, in principle, to any system, whether biological or artificial or a combination of the two.

One novel and important aspect of our approach is that it proposes that inputs to and outputs from the categorization or conceptual system (i.e. the hierarchy of internal QRFs) are encoded on the same boundary, $\mathscr{B}_{in}$ in Fig. 4. This architecture, which is a natural fit with predictive-processing models such as the FEP framework—or indeed with any framework that respects the inherently bidirectional nature of all physical interactions—obviates any need for duplicate categorization or conceptual systems to interpret what is displayed on the inner screen, a commitment that drives critiques, e.g. that of Maturana and Varela (1980), of “representationalism” in traditional models of cognition and consciousness.

While this question is less central to our proposal, placing the inner screen that encodes experience at the whole-system level as close to the informational periphery as possible—noting that the 3D anatomy of this periphery may be very complicated—avoids the potential regress of internal “perceptual” processing, and attendant risk of circularity, of “theater of consciousness” models that postulate an “internal observer” along the lines of Baars (2005); see Safron (2021a), Safron *et al.* (2022) for a construction of “internal observers” as constrained self-models. Encoding whole-system level experience peripherally has an immediate and powerful consequence: because the same low-level QRFs are used for both environmentally-driven and imaginative experience, the inputs to and outputs from the executive/metacognitive system are not experienced at the whole system level unless they are encoded as imaginative content by the lower-level categorization or conceptual system, with encoding by inner speech an obvious example. Again, this consequence is consistent with the “flat mind” hypothesis of Chater (2018), and is supported by the evidence adduced in support of that hypothesis. It is inconsistent with any models that localize whole-system level experience at the boundary of the executive/metacognitive system, as both GW models and some varieties of higher-order thought (HOT; Brown *et al.* (2019)) theory are easily interpreted as doing—though the condition that experiences can only be of what is inscribed on (relatively peripheral) systemic boundaries is *a priori* consistent with there being internal determinants of *whether* a system experiences a given inscribed content, as in versions of HOT that take relevant higher-order states to merely “index” first-order content without duplicating it (Lau, 2019). Similarly, our proposal is inconsistent with models according to which the *content* of experience is suffused throughout the system, as it appears to be in IIT 3.0 (Balduzzi and Tononi, 2009) and the recent models of Bennett, Welsh and Ciaunica (2024) and Laukkonen and Chandaria (2024), though it is consistent with the *processing* of experience being fully distributed.

Our model is novel in its treatment of the role of TFE flow in regulating cognition and hence experience, explicitly distinguishing the content of experience, whether interoceptive or exteroceptive, from its enabling condition, i.e. from adequate TFE flow to power cognition. It also explicitly represents homeostatic interoception as refractory to executive/metacognitive control, consistent with models of emotional experience as primary.

Like the FEP itself (Friston, 2019), the model here depends primarily on mathematical and physical assumptions, particularly on the quantization of information that underpins quantum theory, the association between information and energy that underpins classical thermodynamics, and the local causality that underpins Special Relativity. The model is, therefore, primarily a formal demonstration that the FEP can, in fact, account for imaginative experience. The primary empirical assumption of the model is that environmentally-driven and imaginative experiences are processed by the same QRFs, and hence the same physical or anatomical structures in an agent’s body. It is this assumption that entails the peripheral encoding of imaginative experience, and hence the need for the inner screen $\mathscr{B}_{in}$. Any evidence that pathways implementing imagination are distinct from, or use conceptual or categorical representations not available to, environmentally-driven sensory pathways, would be evidence against this model. We acknowledge that direct tests of the model may not be feasible; while neuroimaging methods reveal the processing pathways involved in sensory experience, they do not reveal the locus of encoding of experiential contents. While it is plausible to identify the informational boundary of a cell with its membrane, or of a brain with its sensory interfaces, including those in the brainstem, the informational boundary of the “I” that reports subjective experiences is notoriously undefined. Identifying the anatomical locus of the proposed $\mathscr{B}_{in}$ is, therefore, not at all straightforward; as noted earlier, the anatomy of $\mathscr{B}_{in}$ may be very complex. That said, understanding both the transition from Fig. 3 architectures to Fig. 4 architectures in phylogeny and the large trait diversity in imaginative experience of humans will require substantial advances in functional neuroscience, in particular in mapping the anatomical correlates of system-scale informational boundaries. We hope the work presented here will be useful in guiding this process.

### Acknowledgements

The authors thank Philippe Blouin, Guillaume Dumas, Jonas Mago, David Rudrauf, Grégoire Sergeant, Lars Sandved Smith, Anil Seth, Toby St Clere Smithe, Jeff Yoshimi, Robert Worden, the members of the CompPheno Fridays discussion group, and the other members of VERSES for valuable comments on early versions of this work, and for useful discussions that shaped the contents of the paper. Special thanks are due to Jakob Hohwy, Mark Solms, Wanja Wiese, and Ken Williford.

### Conflict of interest

None declared.

### Funding

The authors are grateful to VERSES for supporting the open access publication of this paper. BK acknowledges the support of a grant from the John Templeton Foundation (61780). KF is supported by funding for the Wellcome Centre for Human Neuroimaging (Ref.: 205103/Z/16/Z) and a Canada-UK Artificial Intelligence Initiative (Ref.: ES/T01279X/1). The opinions expressed in this publication are those of the author(s) and do not necessarily reflect the views of the John Templeton Foundation.

## Footnotes

1 This statement is, and is intended to be, very general, applying to “inert” systems such as rocks (Friston *et al.*, 2022) or even elementary particles as well as to complex, self-sustaining (i.e. functionally closed), adaptive systems such as organisms or ecosystems. Distinctions between such systems can be constructed within the FEP framework, in terms of computational architecture and capabilities as discussed below; again see Friston (2019) or Friston *et al.* (2022) for overviews.

2 There are many nuanced senses of “free will” that go well beyond the straighforward “physical” idea of freedom from local determinism implied by the Conway-Kochen theorem, which is indeed required for separability between system and environment (Fields *et al.* 2022); see e.g. O’Connor (2022) for review. For the purposes of this paper, freedom from local determinism is sufficient.

3 This reading is highly deflationary, as it treats “experience” as a simple synonym for the inscription of physical traces, by some process or other, on the boundary. Some consciousness researchers explicitly define “experience” or “consciousness” in more restrictive terms (e.g. requiring an experienced sense of self or “awareness of awareness”) or by appealing to intuitions that restrict the range of conscious entities. Such questions are beyond the present scope; see Fields and Levin (2025) for related discussion and Andrews *et al.* (2025) for a recent perspective from biology.

4 The strength of this statement becomes evident when it is applied to human observers, particularly experimental scientists. Scientists observe systems only via their own MBs. The consequences of this fact for the epistemology of science are of obvious interest, but are beyond the present scope; see Moore (1956) for an early formalization.

5 It should be noted that the term “holography” has been used in two different (but conceptually related) ways in the literature, which should be distinguished from each other. On the one hand, the term “holography” refers to a set of techniques allowing for the reconstruction of a three-dimensional image by exploiting the interference patterns of multiple two-dimensional wavefronts. The term “holographic,” as used in connection with the holographic principle in physics, retains the essential idea that the information content of a bulk can (from the point of view of an observer) in principle be encoded on a lower-dimensional surface, but without any commitment to encoding via interference patterns. The former sense of holography was in play in previous theories of consciousness, e.g. the holonomic brain theory (Pribram *et al.*, 1991); it is the latter sense of holography that is relevant here.

6 The holographic principle is sometimes stated as the claim that the bulk can contain no more information than is encodable on the boundary, leaving out the qualifier “externally-observable or accessible.” While in some settings this is innocent, in others it introduces errors; see, e.g. the discussion of Fig. 19 (Wheeler’s “bag of gold”) in (Almheiri *et al.*, 2021). The “bag of gold” is a large system with a small boundary and hence a weak interaction with its environment; it is therefore a good model for an organism. If *all* the information in a system is exposed on its boundary, it cannot have the “internal states” required by the FEP.

7 It should be noted that this carving up of MBs into “thermodynamic” and “informative” sectors inherits the observer-relativity of any description of systems in terms of information-processing (Horsman *et al.*, 2014; Fields *et al.*, 2024). While certain subsystems of biological agents are clearly specialized for certain types of action or for the integration of sensory information via specific channels, there is a generic reading of “sensory” and “active” states that licenses a simpler duality between physical and information-theoretic free energy dynamics (Friston *et al.*, 2020; Kiefer, 2020). For example, as implied in the Introduction, the dissipation of heat may be regarded as a trivial, uninteresting form of “action” on the environment.

8 The environmental feedback that enables the experience of actions may be very fast; one can see one’s hand moving via feedback at the speed of light. Even neural reafferance loops are “environmental” for a brain from an informational-boundary perspective.

9 Later on, we will discuss trait variability in the capacity for imaginative experience. Perhaps the most sane thing to say about the extension of *S* is that it, too, may vary, not only across species but (to a lesser extent) both across individuals and perhaps across time in the same individual. Philosophical debates about the richness or paucity of perceptual experience, for example, may appear intractable in part because different parties to the debate experience the contents of inner screens that exist at different computational distances from $\mathscr{B}_{out}$, and thus correspond to different degrees of multimodal sensory integration and thus to genuinely different experienced contents. At the least, we have as yet considered no principles that rule out such variability.

10 Presumably, aphantasia would impact capacities that directly leverage mental imagery—for example, the ability to draw or paint from memory, which appears in a rather extreme form in artists such as Stephen Wiltshire, who can apparently simply copy inner iconic representations onto canvas just as one might draw from a photograph.
